# InAsSb single crystal with compositional homogeneity grown in outer space

**DOI:** 10.1093/nsr/nwaf208

**Published:** 2025-05-21

**Authors:** Jidong Huang, Zhigang Yin, Jinliang Wu, Xiuhong Pan, Meibo Tang, Xuechao Liu, Xingwang Zhang

**Affiliations:** State Key Laboratory of Semiconductor Physics and Chip Technologies, Institute of Semiconductors, Chinese Academy of Sciences, China; Center of Materials Science and Optoelectronics Engineering, University of Chinese Academy of Sciences, China; State Key Laboratory of Semiconductor Physics and Chip Technologies, Institute of Semiconductors, Chinese Academy of Sciences, China; Center of Materials Science and Optoelectronics Engineering, University of Chinese Academy of Sciences, China; State Key Laboratory of Semiconductor Physics and Chip Technologies, Institute of Semiconductors, Chinese Academy of Sciences, China; Shanghai Institute of Ceramics, Chinese Academy of Sciences, China; Shanghai Institute of Ceramics, Chinese Academy of Sciences, China; Shanghai Institute of Ceramics, Chinese Academy of Sciences, China; State Key Laboratory of Semiconductor Physics and Chip Technologies, Institute of Semiconductors, Chinese Academy of Sciences, China; Center of Materials Science and Optoelectronics Engineering, University of Chinese Academy of Sciences, China

## Abstract

This work reports the growth of an InAsSb single crystal containing 6.7 mol% Sb aboard the China Space Station, achieving superior crystallinity and compositional homogeneity compared to its terrestrial counterpart.

III-V ternary compound semiconductors have emerged as a versatile platform for a wide range of applications, including next-generation photodetectors, post-Moore era high-speed electronics, and advanced quantum computing architectures, due to their ability to flexibly tune the bandgap through compositional adjustments [1]. However, for III-V alloy systems with substantial lattice mismatches (>6%) between the end binaries—such as InAs-InSb, GaAs-InAs, and GaSb-InSb—a compositional deviation of <5 mol% relative to the binary seed crystal is necessary to ensure bulk single crystal growth [2]. In addition to the lattice mismatch between the crystal and the seed, the buoyancy-driven convection, which leads to compositional fluctuations and non-uniform cooling across the melt, also contributes to this critical threshold. Although the growth of III-V ternary alloys has been conducted aboard the International Space Station (ISS) and the TianGong-2 space lab [3,4], full-scale single crystal growth with compositions exceeding the aforementioned empirical threshold has yet to be achieved. The China Space Station (CSS), with its enhanced capability for long-duration microgravity research, has recently demonstrated remarkable breakthroughs in both crystal growth and *in-situ* materials processing [5].

In this work, InAs_1−x_Sb_x_ (InAsSb) with a Sb composition up to 6.6 mol% was grown on a (111)-oriented InAs seed using the vertical gradient freeze (VGF) method aboard the CSS on March 21, 2024, as illustrated in Fig. 1A. The microgravity (μg) experiment was conducted following this procedure: (i) the sandwich starting material of InAs-seed/InSb/InAs-feed was heated to 720°C (at the InAs-seed/InSb interface) within 4 h; (ii) the sample cartridge was held at 720°C for 3 h to establish a stable compositional gradient in the melt, due to the dissolution of InAs (melting point: 942°C) into molten InSb; (iii) the temperature was gradually decreased at a rate of 0.4°C/h while maintaining a constant temperature gradient of 40°C/cm for 24 h; and (iv) the sample cartridge was cooled to room temperature over a period of 9 h. In order to facilitate subsequent characterizations, the obtained μg ingot (Fig. 1B) was bisected into two halves, with the longitudinal section (parallel to the axial direction) aligned along the (1-10) plane of the InAs seed. For comparative analyses, we also performed InAsSb growth under terrestrial (1 g) conditions, using otherwise identical parameters but with a prolonged growth time of 60 h.

**Figure 1.**
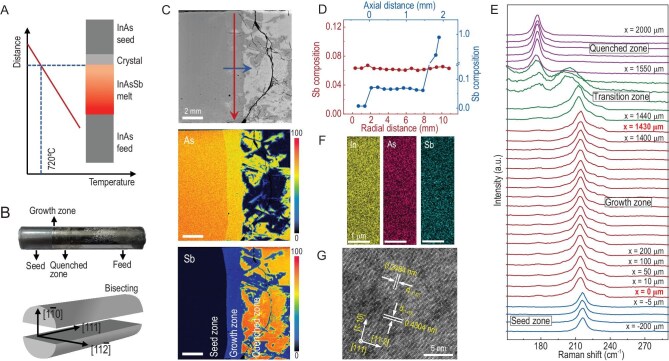
InAsSb single crystal grown under microgravity conditions. (A) Schematic of InAsSb space growth; a nominal temperature of 800°C is calibrated to be 720°C near the InAs-seed/InSb interface at the onset of growth. (B) Photograph of the μg crystal and schematic of ingot dissection. (C) EPMA characterizations; from top to bottom: backscattered electron image, and elemental mapping of As and Sb. (D) EPMA line scans along the radial (red) and axial (blue) directions. (E) Linear scan of Raman spectra along the axial direction. (F) TEM-EDS mapping. (G) High-resolution TEM image.

Figure 1C shows the electron probe microanalysis (EPMA) elemental maps collected on the longitudinal section of the μg ingot. It reveals the μg sample consists of three parts: the seed, growth zone, and quenched zone. Over a total growth length of ∼1.5 mm and a crystal diameter of ∼11 mm, the μg crystal demonstrates excellent compositional homogeneity, with no noticeable striations being visible. Quantitative analysis reveals an average Sb composition *x* of 0.066, with compositional fluctuations less than ±0.5 mol%. These findings are further corroborated by the EPMA line-scan profiles along both axial and radial directions (Fig. 1D). Note that in previous experiments, the obtained InAsSb bulk crystals possessed a graded composition distribution along the axial direction [6]. In line with the compositional uniformity demonstrated by EPMA, the transverse optical (TO) phonon Raman peaks of InAsSb remain constant at ∼214.3 cm^−1^ throughout the entire growth zone, exhibiting a characteristic redshift of ∼2 cm^−1^ compared to the TO mode (216.3 cm^−1^) of the InAs seed (Fig. 1E). Similarly, the macroscopic Raman peak uniformity within the InAsSb crystal is also confirmed by the linear scan along the radial direction, as revealed in Fig. S1. The high resolving power of the Raman spectrometer also enables clear identification of a distinct transition zone between the growth and quenched zones, which corresponds to the crystallization process during the rapid temperature drop from 720°C to 525°C (the melting point of InSb) near the interfacial region.

Notably, the μg InAsSb sample possesses a sharply defined, nearly planar seed/crystal interface (Fig. 1C). This contrasts with the much more convex interfacial morphology observed in the ISS-grown SiGe crystals [7]. In VGF crystal growth, there are three distinct types of solid/melt interface morphologies: planar, convex and concave. It has been well established that both the concave and convex interfaces induce nonuniform stress along the radial direction, thereby resulting in high defect densities in the crystals [2]. Moreover, the solid/melt interface represents the isotherm corresponding to the melting point of the material, and both the concave and convex morphologies have a strong influence on the radial compositional profile of the InAsSb ternary crystal. The well controlled solid/liquid interface morphology under microgravity conditions enables a remarkably uniform transfer of heat and mass along the axial direction, giving rise to an improved interfacial quality of the InAsSb crystal.

In order to elucidate the structural characteristics, we also carried out transmission electron microscopy (TEM) characterizations on the μg sample. As shown in Fig. 1F, the energy dispersive X-ray spectroscopy (EDS) mapping reveals a uniform distribution of the elements In, As, and Sb at the micrometer level. Moreover, no impurity phases or inclusions are discernible in the InAsSb sample. Figure 1G presents a typical high-resolution TEM image, which shows the layer spacings of 4.304 Å and 2.484 Å along the [1-10] and [11-2] directions, respectively. These findings reveal that the TEM specimen (Fig. 1B) has a zone axis of [111], providing robust evidence on the epitaxial growth of InAsSb bulk crystal on the InAs (111) seed. The lattice constant of the InAsSb crystal, calculated as *a* = 6.087 Å using Vegard's law [8], corresponds to an Sb composition of *x* = 0.068, consistent with the EPMA results in Fig. 1C and D.

Electron backscatter diffraction (EBSD) analyses were performed on the transverse section (perpendicular to the axial direction), and the results further corroborate that the μg sample is a (111)-oriented single crystal (Fig. S2A). In sharp contrast, although the terrestrially grown InAsSb sample predominantly exhibits (111)-orientation, domains with alternative crystallographic orientations are clearly resolvable (Fig. S2B), revealing its polycrystalline nature. Also notable is that the μg InAsSb crystal has a full width at half-maximum (FWHM) of the Raman TO peak comparable to the InAs seed, and substantially narrower than its terrestrial counterpart (Fig. S3). In addition, although the μg crystal remains void-free, spherically shaped macroscopic voids with size up to ∼3 mm are clearly present in the 1 g sample (Fig. S4). In general, the formation of voids is always accompanied by the occurrence of multigrain growth [9], as grain boundaries serve as efficient nucleation sites for voids. All these findings conclusively establish that the μg InAsSb crystal is single-crystalline with crystallinity comparable to the InAs seed, whereas its terrestrial counterpart exists in a polycrystalline form. It is well known that a high growth rate can lead to constitutional supercooling near the solid/melt interface, which promotes polycrystalline growth [2]. Additionally, buoyancy-driven convective flow can cause severe composition fluctuations near the solid/melt interface, further contributing to nonuniform growth and the formation of multiple grains. It is the combination of a low growth rate (∼0.06 mm/h) and the suppression of buoyancy-induced convection that leads to the growth of InAsSb single crystal under microgravity conditions. By contrast, although terrestrial growth exhibits an even lower growth rate (∼0.04 mm/h), the strong convection flow, as demonstrated by recent numerical simulations [10], results in the formation of a polycrystalline sample.

In conclusion, we have successfully grown a InAsSb single crystal aboard the CSS, achieving a Sb composition up to 6.6 mol%. Our findings challenge the notion that in III-V alloy systems with lattice mismatches exceeding 6% between the end binaries, single crystal growth on a binary substrate is only feasible when the composition deviation is <5 mol%. In contrast, only polycrystalline growth was observed under terrestrial conditions. Our results indicate that the nearly complete absence of buoyancy-driven convection in microgravity plays a crucial role in suppressing growth non-uniformity and multi-oriented domain formation. The microgravity-grown crystal features a sharp, nearly planar seed/crystal interface and is free of macroscopic voids. In addition, it demonstrates compositional uniformity on both macroscopic and micrometric levels, with composition fluctuations of less than ±0.5 mol%. The high-quality InAsSb single crystal obtained here reveals that the microgravity environment provides a unique platform for synthesizing materials that cannot be grown terrestrially.

## Supplementary Material

nwaf208_Supplemental_File

## References

[1] Pendharkar M, Zhang B, Wu H et al. Science 2021; 372: 508–11. 10.1126/science.aba5211 33858990

[2] Dutta PS . Springer Handbook of Crystal Growth. Berlin: Springer, 2010, 281–325. 10.1007/978-3-540-74761-1_10

[3] Yu J, Inatomi Y, Nirmal Kumar V et al. npj Microgravity 2019; 5: 8. 10.1038/s41526-019-0068-1 30963108 PMC6443717

[4] Nirmal Kumar V, Arivanandhan M, Rajesh G et al. npj Microgravity 2016; 2: 16026. 10.1038/npjmgrav.2016.26 28725736 PMC5515529

[5] Dixit VK, Bansal B, Venkataraman V et al. Appl Phys Lett 2002; 81: 1630–2. 10.1063/1.1504163

[6] Wang H, Liao H, Hu L et al. Adv Mater 2024; 36: 2313162. 10.1002/adma.202313162 38461368

[7] Arai Y, Kinoshita K, Tsukada T et al. Cryst Growth Des 2018; 18: 3697–703. 10.1021/acs.cgd.8b00544

[8] Wickramaratne D, Mazin II. Nat Commun 2022; 13: 2376. 10.1038/s41467-022-29213-8 35501318 PMC9061790

[9] Nishijima Y, Nakajima K, Otsubo K et al. J Cryst Growth 1999; 197: 769–76. 10.1016/S0022-0248(98)00925-7

[10] Jin X, Xu S, Wang B et al. Phys Fluids 2025; 37: 037115. 10.1063/5.0260217

