# Supplementary material for: InAsSb single crystal with compositional homogeneity grown in outer space
Source: Natl Sci Rev. 2025 May 21;12(7):nwaf208. doi: 10.1093/nsr/nwaf208 (PMC12166300; doi:10.1093/nsr/nwaf208)
Supplement: nwaf208_Supplemental_File [file nwaf208_supplemental_file.pdf]

Supplementary Material

InAsSb single crystal with compositional homogeneity grown in outer space

Jidong Huang[1,2], Zhigang Yin[1,2,*], Jinliang Wu[1], Xiuhong Pan[3], Meibo Tang[3], Xuechao Liu[3] and Xingwang Zhang[1,2,*]

[1]State Key Laboratory of Semiconductor Physics and Chip Technologies, Institute of Semiconductors, Chinese Academy of Sciences, Beijing 100083, China;

[2]Center of Materials Science and Optoelectronics Engineering, University of Chinese Academy of Sciences, Beijing 100049, China;

[3]Shanghai Institute of Ceramics, Chinese Academy of Sciences, Shanghai 201899, China

*Corresponding authors. E-mails: yzhg@semi.ac.cn; xwzhang@semi.ac.cn

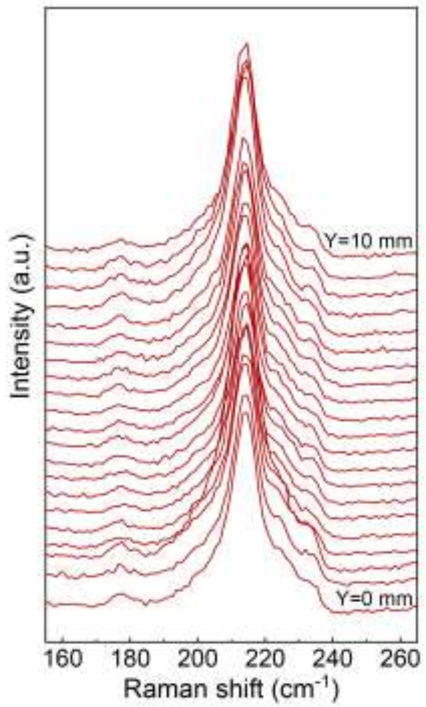

Figure S1. Linear scan of the Raman spectra along the radial direction for the μg sample.

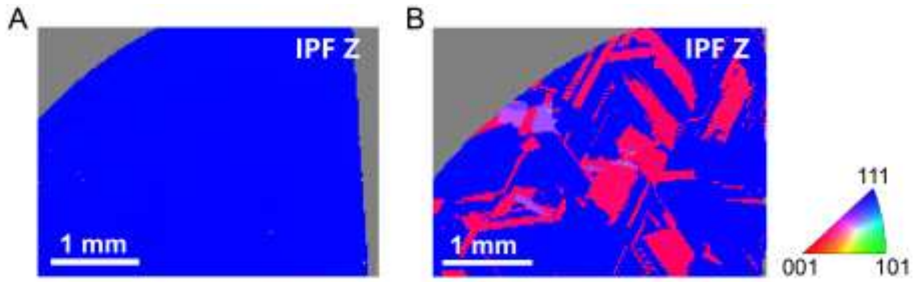

Figure S2. EBSD out-of-plane (Z) inverse pole figures for the μg (A) and 1g (B) samples.

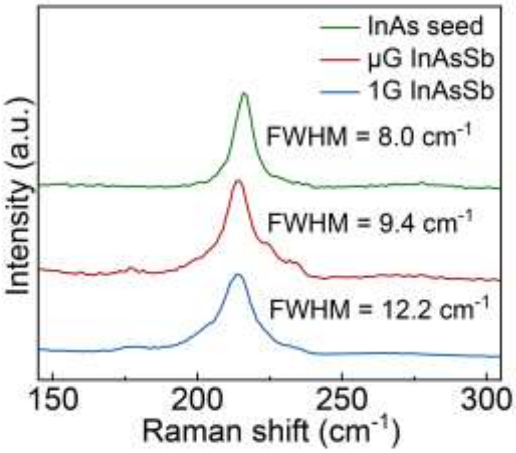

Figure S3. Comparative illustration of the Raman spectra for the μg sample, 1g sample and the seed.

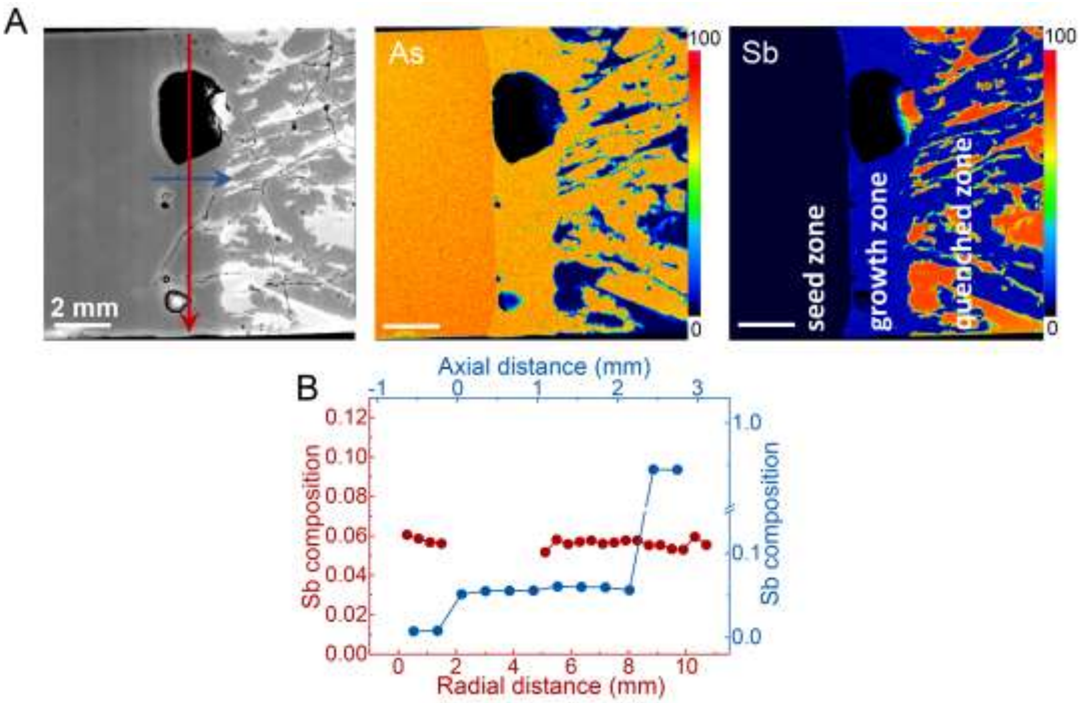

Figure S4. (A) EPMA characterizations for the 1g sample; From left to right: backscattered electron image, and elemental mapping of As and Sb; macroscopic voids are clearly visible. (B) EPMA line scans along the radial (red) and axial (blue) directions; the average Sb composition is 5.7 mol%. A growth time of 60 hours has been adopted to facilitate better comparative characterizations, and the growth rate is determined to be ~0.04 mm/h. The results obtained here are in qualitatively agreement with previous reports that the growth rate under space microgravity is higher than that on the ground [1-3].

REFERENCES

1. Hayakawa Y, Okano Y, Hirata A *et al*. *J Cryst Growth* 2000; **213**: 40-50.

2. Nirmal Kumar V, Arivanandhan M, Rajesh G *et al*. *npj Microgravity* 2016; **2**: 1-7.

3. Jin X, Xu S, Wang B *et al*. *Phys Fluids* 2025; **37**: 037115.
